# Association Between Appetite and Sarcopenia in Patients With Mild Cognitive Impairment and Early-Stage Alzheimer's Disease: A Case-Control Study

**DOI:** 10.3389/fnut.2018.00128

**Published:** 2018-12-18

**Authors:** Ai Kimura, Taiki Sugimoto, Shumpei Niida, Kenji Toba, Takashi Sakurai

**Affiliations:** ^1^Center for Comprehensive Care and Research on Memory Disorders, National Center for Geriatrics and Gerontology, Obu, Japan; ^2^Medical Genome Center, National Center for Geriatrics and Gerontology, Obu, Japan; ^3^Department of Cognitive and Behavioral Science, Nagoya University Graduate School of Medicine, Nagoya, Japan; ^4^Department of Community Health Sciences, Graduate School of Health Sciences, Kobe University, Kobe, Japan; ^5^Japan Society for the Promotion of Science, Tokyo, Japan

**Keywords:** sarcopenia, appetite, mild cognitive impairment, early-stage Alzheimer's disease, satiety, gustatory function

## Abstract

**Background:** Sarcopenia is frequently seen in patients with mild cognitive impairment (MCI) and early-stage Alzheimer's disease (AD). While appetite loss and physical inactivity, which are also frequently seen in dementia, appear to contribute to sarcopenia, to date, no study has investigated this association.

**Objective:** The aim of this study was to examine factors associated with sarcopenia, including appetite and physical activity, in patients with MCI and early-stage AD.

**Methods:** The study subjects comprised 205 outpatients (MCI, *n* = 151; early-stage AD, *n* = 54) who were being treated at the Memory Clinic, National Center for Geriatrics, and Gerontology and had a Mini-Mental State Examination (MMSE) score of 21 or higher. All subjects were assessed for appetite by using the Council on Nutrition Appetite Questionnaire (CNAQ). Confounding variables assessed included physical activity, activities of daily living, mood, body mass index (BMI), nutritional status, and medications. Sarcopenia was defined as low muscle mass and low handgrip strength or slow gait speed. Multivariate logistic regression analyses were performed with adjustment for age, gender, education, and confounding variables to examine the association of sarcopenia with physical activity and appetite. Furthermore, sub-analyses were also conducted to clarify the relationship between CNAQ sub-items and sarcopenia.

**Results:** The prevalence of sarcopenia among the subjects was 14.6% (*n* = 30). Patients with sarcopenia had lower CNAQ scores (those with sarcopenia, 26.7 ± 3.5; those without, 29.1 ± 2.5). Multivariate analysis showed that BMI (odds ratio [OR], 0.675; 95% confidence interval [CI], 0.534–0.853), polypharmacy (OR, 4.489; 95% CI, 1.315–15.320), and CNAQ (OR, 0.774; 95% CI, 0.630–0.952) were shown to be associated with sarcopenia. Physical activity was not associated with sarcopenia. Of the sub-items of the CNAQ, appetite (OR, 0.353; 95% CI, 0.155–0.805), feeling full (OR, 0.320; 95% CI = 0.135–0.761), and food tastes compared to when younger (OR, 0.299; 95% CI, 0.109–0.818) were shown to be associated with sarcopenia.

**Conclusions:** These results suggest that appetite could be a modifiable risk factor for sarcopenia in patients with MCI and early-stage AD. A comprehensive approach to improving appetite may prove effective in preventing sarcopenia.

## Introduction

Frailty is construed as a consequence of age-related decline in many physiological systems, resulting in vulnerability to sudden adverse changes in health status triggered by minor stressor events (1). Frailty is thus thought to represent a combination of problems in different domains of human functioning, such as physical, psychological, and social domains (2). Since frailty is known to be associated with adverse outcomes (1), prevention of onset of frailty, as well as care of the elderly with frailty, remains the most urgent of challenges in clinical practice.

Of the three different domains of frailty, sarcopenia is attracting attention as a cause of physical frailty (1) and is defined as low skeletal muscle mass and low muscle strength or low physical performance (3). Factors associated with sarcopenia are multifaceted and include aging, inappropriate nutrition, low physical activity, chronic inflammation, and sex hormones (3). Of these, in particular, nutrition and exercise are known to be important modifiable risk factors.

Sarcopenia increases with age, but it is more prevalent in patients with dementia. It is also reported that the prevalence of sarcopenia is higher among patients with mild cognitive impairment (MCI) and Alzheimer's disease (AD) than among older people with normal cognition (4) and that older age, low body mass index (BMI), and low vitality are associated with sarcopenia in patients with MCI and AD. Low vitality, which overlaps with apathy, is one of the most common neuropsychiatric symptoms among patients with dementia (5), likely affecting appetite and physical activity. In fact, several studies demonstrated that appetite and physical activity are decreased in patients with dementia (6–8). Therefore, appetite loss and low physical activity appear to be key factors associated with sarcopenia in patients with dementia. However, no study investigated the association of sarcopenia with appetite or physical activity. Furthermore, there are no studies focused on patients with MCI and early-stage AD.

The aim of this study was to examine factors associated with sarcopenia, including appetite and physical activity, in patients with MCI and early-stage AD. A previous study showed that sarcopenia is associated with impaired activities of daily living (ADL) in early-stage AD (9). Therefore, identification of factors associated with sarcopenia in patients with dementia may prove helpful in preventing loss of their independence leading to worsening of not only their quality of life (QOL) but their caregivers' QOL.

## Materials and Methods

### Subjects

The subjects of this study were outpatients (aged 65–89 years) who presented to the Memory Clinic at the National Center for Geriatrics and Gerontology (NCGG) of Japan during the period from February 2015 to January 2017. For the present analysis, we included those with a Mini-Mental State Examination (MMSE) score of 21 or higher who were diagnosed with MCI, and AD, given that a MMSE score of 21 or higher may be used as a surrogate measure for the Clinical Dementia Rating for staging of questionable (0.5) to mild (1.0) dementia in AD (10). MCI and AD were diagnosed based on the criteria of the National Institute on Aging-Alzheimer's Association Workgroups (11, 12). Excluded from the study were those who had serious disease (cardiac, pulmonary, hepatic, and kidney disease), inflammatory disease (e.g., rheumatoid arthritis), cancer, and/or psychiatric disease other than dementia, as well as those who were bedridden.

The study included a total of 333 patients who had completed assessments for grip strength, gait speed, and skeletal muscle mass index (SMI). Finally, a total of 205 patients were available for analysis, after excluding 128 patients with missing data for any of the variables examined (age, gender, cognitive status, ADL, depressive mood, vitality, BMI, nutritional status, smoking status, comorbid conditions, or polypharmacy) that are reported to be associated with sarcopenia (3, 4, 13–16). Of these 205 subjects, 151 and 54 patients were diagnosed as having MCI and AD, respectively.

The study protocol was approved by the Ethics/Conflict of Interest Committee at the NCGG. Written informed consent was obtained from all patients before their study participation.

### Measurements

#### Appetite

All patients were assessed for appetite by using the Council on Nutrition Appetite Questionnaire (CNAQ) (17), which consists of the following 8 questions: appetite, feeling full, feeling hunger, food tastes, food tastes compared to when younger, meal frequency per day, feel sick or nauseated when eating, and usual mood. The subjects and their families (or caregivers) were requested to respond to each question using 1–5 ordinal scales (Likert scales). The CNAQ total score ranges from 8 (worst) to 40 (best) points, while lower scores indicate deterioration in appetite. The reliability and validity of the Japanese version of the CNAQ have previously been established (18).

#### Physical Activity

We evaluated physical activity using the following questions: (1) “Do you engage in moderate-level physical exercise or sports?” and (2) “Do you engage in low-level physical exercise?” Subjects were considered to be inactive if they answered “No” to both these questions (19).

#### Muscle Mass and Strength

Appendicular muscle mass was measured by bioelectrical impedance analysis (BIA) using a Tanita body composition analyzer (MC-180; Tanita Corp., Tokyo, Japan). Skeletal muscle mass index (SMI) was calculated as appendicular muscle mass divided by height squared (kg/m^2^) (20). Muscle strength was assessed by hand grip strength, measured with a digital force gauge (ZP-500N; Imada, Toyohashi, Japan) (21).

#### Physical Performance

Physical performance was measured by the gait speed. Gait speed was assessed during normal walking, with the participant instructed to walk at his or her preferred speed.

### Definition of Sarcopenia

Sarcopenia was defined according to the consensus of the Asian Working Group for Sarcopenia criteria (22), which includes three components: low handgrip strength (<6 kg for men and <8 kg for women), slow gait speed (<0.8 m/s on 2.4-m walking at the usual pace), and low muscle mass as assessed with the SMI (7.0 kg/m^2^ for men and 5.7 kg/m^2^ for women as measured by BIA). The presence of sarcopenia was defined as low muscle mass and low handgrip strength or slow gait speed.

### Other Assessments

Demographic data on the subjects' age, years of education, marital status, smoking status, drinking status, living situation, and economic conditions were obtained from their clinical charts. The subjects were assessed for the presence of chronic disease (diabetes mellitus, hypertension, dyslipidemia, cardiac disease, and pulmonary disease) and the number of medications they were on. Polypharmacy was defined as five or more regularly prescribed drugs (23). Nutritional status was assessed based on the Mini-Nutritional Assessment Short-Form (24), BMI, and serum levels of albumin, total protein, and 25-hydroxy vitamin D. Global cognitive function was examined by using the MMSE, with the scores ranging from 0 to 30 (25). Basic ADL and instrumental ADL were evaluated by using the Barthel Index (26) and the Lawton Index (27), respectively. Vitality was assessed by using the Vitality Index (28). This questionnaire included five items (waking pattern, communication, feeding, on and off toilet, and rehabilitation and other activities) and was answered by the subjects' caregivers. With the total score of this questionnaire ranging between 0 and 10 points, it is judged that the closer the score comes to 0 points, the lower the vitality. All subjects were evaluated for depressive mood by using the 15-item Geriatric Depressive Scale (29). All subjects were also assessed for inflammatory conditions based on serum C-reactive protein concentrations.

### Statistical Analysis

The study examined the prevalence of sarcopenia in the study subjects. To examine the association of sarcopenia with physical activity and appetite, differences in physical activity, CNAQ, and clinical profiles between the subjects with and without sarcopenia were analyzed in univariate analyses using the Mann-Whitney *U* test for continuous variables, and the chi-squared test for categorical variables. In multivariate analyses, logistic regression analysis was performed incorporating physical activity, CNAQ, and all variables associated with *P* values < 0.10 in univariate analyses, with adjustment for the following confounding factors shown to be associated with sarcopenia in previous studies (3, 4, 13–16): sex, age, education, cognition, ADL, depression, vitality, nutritional status, physical performance, smoking status, comorbidities, and polypharmacy. In order to examine the association between sarcopenia and appetite in detail, logistic regression analyses were conducted by incorporating the sub-items of the CNAQ.

Data are presented as mean ± standard deviation unless stated otherwise. A value of *P* < 0.05 was considered to indicate statistical significance, and adjusted odds ratios (OR) and their 95% confidence intervals (CIs) were reported.

All analyses were carried out by using the Japanese version of SPSS for Windows version 23.0 (IBM Corporation, Armonk, NY, USA).

## Results

The clinical profile of the subjects with and without sarcopenia is shown in Table 1. The subjects' mean age was 77.2 ± 5.1 years, and 130 (63.4%) were female. The prevalence of sarcopenia was 14.6%. The mean SMI, grip strength, and gait speed were 6.4 ± 1.0 kg/m^2^, 23.3 ± 7.3 kg, and 1.0 ± 0.2 m/s, respectively.

**Table 1:** Clinical characteristics of those with sarcopenia and those without.

	**Overall *n* = 205**	**Those with sarcopenia *n* = 30**	**Those without sarcopenia *n* = 175**	***P* value**
Female, *n* (%)	130 (63.4)	22 (73.3)	108 (61.7)	0.305
Age, years	77.2 ± 5.1	79.4 ± 5.0	76.9 ± 5.1	0.011
AD, *n* (%)	54 (26.3)	8 (26.7)	46 (26.3)	1.000
Education, years	10.8 ± 2.3	10.3 ± 2.0	10.9 ± 2.4	0.233
Marital status, *n* (%), *n* = 203				1.000
Never married	3 (1.5)	0 (0.0)	3 (1.7)	
Married	200 (98.5)	30 (100.0)	170 (98.3)	
Living alone, *n* (%), *n* = 204	24 (11.8)	5 (16.7)	19 (10.9)	0.362
Need for financial support, *n* (%), *n* = 199	12 (5.9)	0 (0.0)	12 (6.9)	0.221
MMSE, score	24.7 ± 2.6	24.4 ± 2.7	24.7 ± 2.6	0.411
Barthel Index, score	98.8 ± 3.7	97.5 ± 5.4	99.0 ± 3.3	0.041
Lawton Index, score	6.1 ± 1.7	5.7 ± 1.6	6.1 ± 1.7	0.197
Male	4.4 ± 0.8	4.3 ± 0.9	4.5 ± 0.8	0.445
Female	7.0 ± 1.3	6.3 ± 1.4	7.1 ± 1.2	0.003
Vitality Index, score	9.4 ± 1.0	9.1 ± 1.1	9.4 ± 1.0	0.013
GDS-15, score	2.8 ± 2.6	4.0 ± 3.4	2.6 ± 2.3	0.062
SMI, kg/m^2^	6.4 ± 1.0	5.5 ± 0.6	6.6 ± 0.9	<0.001
Grip strength, kg	23.3 ± 7.3	16.7 ± 5.1	24.4 ± 7.0	<0.001
Gait speed, s/m	1.0 ± 0.2	0.9 ± 0.3	1.0 ± 0.2	0.054
Physical activity, inactivity, *n* (%)	53 (25.9)	12 (40.0)	41 (23.4)	0.071
Body Mass Index, kg/m^2^	21.9 ± 3.1	20.0 ± 2.5	22.3 ± 3.1	<0.001
CNAQ, score	28.8 ± 2.8	26.7 ± 3.5	29.1 ± 2.5	<0.001
A. Appetite	3.4 ± 0.8	3.0 ± 0.7	3.5 ± 0.8	0.001
B. Feeling full	3.7 ± 0.6	3.3 ± 0.6	3.7 ± 0.5	<0.001
C. Feeling hunger	2.6 ± 0.9	2.4 ± 0.8	2.6 ± 0.9	0.294
D. Food tastes	3.6 ± 0.7	3.3 ± 0.7	3.6 ± 0.6	0.013
E. Food tastes compared to when younger	3.2 ± 0.6	2.9 ± 0.5	3.2 ± 0.6	0.016
F. Meal frequency per day	3.9 ± 0.3	3.8 ± 0.5	3.9 ± 0.3	0.215
G. Feel sick or nauseated when eating	4.8 ± 0.5	4.7 ± 0.8	4.8 ± 0.4	0.838
H. Usual mood	3.6 ± 0.7	3.3 ± 0.6	3.7 ± 0.7	0.002
MNA-SF, score	11.7 ± 1.9	10.7 ± 1.7	11.8 ± 1.8	0.001
Current smoker, *n* (%)	17 (8.3)	5 (16.7)	12 (6.9)	0.082
Drinking Status, *n* (%)				0.158
Never	126 (61.5)	23 (76.7)	103 (58.9)	
Ethanol <43.2 g/day	75 (36.6)	7 (23.3)	68 (38.9)	
Ethanol ≥ 43.2 g/day	4 (2.0)	0 (0.0)	4 (2.3)	
Polypharmacy, n ≥ 5, *n* (%)	78 (38.0)	19 (63.3)	59 (33.7)	0.004
Anti-dementia drug, *n* (%)	54 (26.3)	8 (26.7)	46 (26.3)	1.000
Comorbid conditions	1.0 ± 1.0	1.1 ± 1.1	1.0 ± 1.0	0.635
Diabetes mellitus, *n* (%)	29 (14.1)	3 (10.0)	26 (14.9)	0.583
Hypertension, *n* (%)	89 (43.4)	14 (46.7)	75 (42.9)	0.696
Dyslipidemia, *n* (%)	71 (34.6)	11 (36.7)	60 (34.3)	0.837
Cardiac disease, *n* (%)	12 (5.9)	4 (13.3)	8 (4.6)	0.080
Pulmonary disease, *n* (%)	9 (4.4)	2 (6.7)	4 (4.0)	0.623
Serum albumin, g/dl, *n* = 156	4.4 ± 0.3	4.3 ± 3.2	4.4 ± 0.3	0.110
Serum total protein, g/dl, *n* = 156	7.3 ± 0.4	7.4 ± 0.4	7.3 ± 0.5	0.235
C-reactive protein, mg/dl, *n* = 150	0.11 ± 0.3	0.12 ± 0.1	0.11 ± 0.3	0.369
25(OH)D, ng/ml, *n* = 113	21.2 ± 9.7	18.4 ± 6.0	21.6 ± 10.1	0.337

*AD, Alzheimer's disease; CNAQ, Council on Nutrition Appetite Questionnaire; GDS-15, 15-item Geriatric Depression Scale; MMSE, Mini-Mental State Examination; MNA-SF, Mini-Nutritional Assessment-Short Form; SMI, Skeletal Muscle mass Index. Data are shown as mean ± standard deviation or n (%). P value are for differences between those with sarcopenia and those without*.

Univariate analyses showed patients with sarcopenia had lower total CNAQ scores (those with sarcopenia vs. those without; 26.7 ± 3.5 vs. 29.1 ± 2.5; *P* < 0.001) and lower CNAQ sub-item scores (appetite loss, feeling full, feel taste of food, tastes of food compared to when younger, and usual mood) than those without sarcopenia. Patients with sarcopenia tended to be inactive (those with sarcopenia, 40.0%; those without, 23.4%; *P* = 0.071). Moreover, compared to those without sarcopenia, patients with sarcopenia were associated with older age (79.4 ± 5.0 vs. 76.9 ± 5.1 years; *P* = 0.011), lower performance in basic ADL (97.5 ± 5.4 vs. 99.0 ± 3.3; *P* = 0.041), lower vitality (9.1 ± 1.1 vs. 9.4 ± 1.0 score; *P* = 0.013), lower grip strength (16.7 ± 5.1 vs. 24.4 ± 7.0 kg; *P* < 0.001), and lower BMI values (20.0 ± 2.5 vs. 22.3 ± 3.1 kg/m^2^; *P* < 0.001) and lower SMI (5.5 ± 0.6 vs. 6.6 ± 0.9 kg/m^2^; *P* < 0.001). The frequency of polypharmacy was significantly high in those with sarcopenia (Table 1).

In multivariate analyses, we conducted multiple logistic regression analyses by incorporating age, education, MMSE, Barthel Index, Lawton Index, 15-item Geriatric Depressive Scale, Vitality Index, BMI, Mini-Nutritional Assessment Short-Form, physical activity, smoking status, comorbid conditions, and polypharmacy, which demonstrated that independent predictors of sarcopenia were BMI (OR, 0.675; 95% CIs, 0.534–0.853), CNAQ total score (OR, 0.774; 95% CIs, 0.630–0.952), and polypharmacy (OR, 4.489; 95% CIs, 1.315–15.320) (Table 2). In analyses focused on the CNAQ sub-items, appetite loss (OR = 0.353, 95% CIs = 0.155–0.805), feeling full (OR = 0.320, 95% CIs = 0.135–0.761), and food tastes compared to when younger (OR = 0.299, 95% CIs = 0.109–0.818) were significantly associated with sarcopenia (Table 3).

**Table 2:** Factors associated with sarcopenia identified in multivariate logistic regression model.

	**B**	**OR (95% CIs)**	***P* value**
Gender	1.001	2.721 (0.380–19.482)	0.319
Age	0.078	1.081 (0.962–1.216)	0.189
Education	0.058	1.060 (0.831–1.351)	0.640
MMSE	0.072	1.075 (0.881–1.311)	0.475
Barthel index	−0.070	0.932 (0.805–1.078)	0.344
Lawton index	−0.400	0.670 (0.382–1.178)	0.164
GDS-15	0.101	1.106 (0.909–1.346)	0.316
Vitality Index	−0.047	0.954 (0.542–1.679)	0.872
Physical activity	0.746	2.108 (0.723–6.146)	0.172
Body mass index	−0.393	0.675 (0.534–0.853)	0.001
CNAQ	−0.256	0.774 (0.630–0.952)	0.015
MNA-SF	0.179	1.196 (0.850–1.684)	0.304
Current smoker	1.029	2.797 (0.592–13.215)	0.194
Comorbid conditions	−0.214	0.808 (0.465–1.403)	0.448
Polypharmacy	1.502	4.489 (1.315–15.320)	0.016

*CNAQ, Council on Nutrition Appetite Questionnaire; GDS-15, 15-item Geriatric Depression Scale; MMSE, Mini-Mental State Examination; MNA-SF, Mini-Nutritional Assessment-Short Form*.

**Table 3:** Association between sarcopenia and the CNAQ sub-items in multivariate logistic regression models.

	**B**	**Adjusted OR (95% CIs)**	***P* value**
Appetite	−1.041	0.353 (0.155–0.805)	0.013
Feeling full	−1.138	0.320 (0.135–0.761)	0.010
Feeling hunger	−0.160	0.852 (0.455–1.596)	0.618
Food taste	−0.340	0.712 (0.335–1.511)	0.376
Food tastes compared to when younger	−1.207	0.299 (0.109–0.818)	0.019
Meal frequency per day	−0.282	0.754 (0.227–2.507)	0.645
Feel sick or nauseated when eating	−0.226	0.798 (0.305–2.086)	0.645
Usual mood	−0.299	0.741 (0.332–1.658)	0.466

*Adjusted for gender, age, education, Mini-Mental State Examination, Barthel Index, Lawton Index, 15-item Geriatric Depression Scale, Vitality Index, body mass index, Mini-Nutritional Assessment-Short Form, physical activity, current smoker, comorbid conditions, and polypharmacy*.

## Discussion

### Summary of Results

The prevalence of sarcopenia in those with MCI and early-stage AD was 14.6%. Independent factors associated with sarcopenia included BMI, polypharmacy, and CNAQ total score, especially appetite loss, feeling full, and food tastes compared to when younger. Physical activity was not associated with sarcopenia in our study subjects with MCI and early-stage AD.

### Factors Associated With Sarcopenia in MCI and Early-Stage AD

In this study, sarcopenia was shown to be independently associated with appetite, in addition to low BMI and polypharmacy that were shown to be associated with sarcopenia in previous studies.

Appetite loss is one of the most frequent behavioral and psychiatric symptoms among patients with AD from early-stage (7, 8) and leads to decreased food intake, which results in weight loss. While causes of appetite loss are multifactorial (30), a recent study reported that appetite was associated with depression, difficulty in maintaining attention while eating, lower vitality, more comorbidities, no use of anti-dementia drugs, and use of psychotropic drugs among patients with cognitive impairment (8). The previous study showed that low vitality was associated with sarcopenia among patients with MCI and AD (4). Although low vitality appears to affect both appetite and physical activity, our study demonstrated that appetite was associated with sarcopenia, but physical activity as evaluated based on the presence or absence of exercise habits was not. The amount of physical and sedentary activity has been reported as a factor associated with sarcopenia among healthy elderly people (31, 32). These conflicting results between the previous studies and ours may be due to differences in the methods used to assess physical activity. Indeed, Akune and colleagues reported that exercise habits in elderly were not related to sarcopenia (33). Moreover, it has been reported that the impact of exercise on sarcopenia treatment varies by frequency and type of exercise (34). Further studies are required to investigate not only exercise habits but also the amount and type of physical activity to corroborate our study findings.

Besides appetite loss, feeling full, and food tastes compared to when younger were associated with sarcopenia. Usually, the onset of satiety represents a response to neural and endocrine factors, such as gastric distension and release of the gut peptide cholecystokinin that are generated during the course of meal ingestion (35). A previous study reported that 28.3 and 27.3% of mild AD patients experienced reduced and increased appetite (7). While the causes of reduced and increased appetite in patients with AD remains unclear, a change in satiety responsiveness may affect their appetite (7, 36). Further studies are needed to clarify changes in satiety among those with dementia.

Gustatory function changes with aging (37). In dementia, some studies reported that the recognition thresholds of the four elements of taste, i.e., sweet, salty, sour, and bitter, are higher in patients with MCI and AD than in healthy subjects (38). In contrast, there is also a study reporting that demented patients and healthy elderly people are similar in gustatory function (39). Given that there are very few studies that focused on gustatory function in patients with dementia, however, patients with dementia require to be further evaluated for changes in gustatory function in future studies.

### Weight Loss and Change in Body Composition Among Patients With Cognitive Impairment

Nutritional problems, notably body weight loss, are frequently seen among those with AD from early-stage, however, the detailed mechanisms are not fully understood. A recent review suggests that weight loss among patients with dementia is caused by a decrease in energy intake due to loss of appetite as well as by an increase in energy expenditure due to increased basal metabolism and behavioral disturbance (40). It has also been reported that skeletal muscle is reduced in early-stage AD, and skeletal muscle mass is associated with brain volume and severity of cognitive impairment (41, 42). Additionally, with the progression of dementia, fat mass is also reduced in patients with dementia (43), while fat mass is generally increased or unchanged in healthy older adults with aging (44, 45). These previous studies indicate a significant link between changes in body composition and cognitive deterioration. Further studies are needed to investigate the natural history and impact of changes in body composition among patients with cognitive impairment.

### Putative Mechanisms of Appetite Loss in Dementia

The hypothalamus plays a critical role in appetite regulation. The energy homeostasis is regulated by satiety and feeding signals in the blood, such as nutrients (e.g., glucose), gut- and neuro-peptides (e.g., ghrelin, cholecystokinin, orexin), and adipocytokines (e.g., leptin), which increase or decrease appetite. Ghrelin stimulates appetite in negative energy balance (35). On the other hand, blood glucose, cholecystokinin, and leptin inhibits appetite in positive energy balance (35). Moreover, given the orexin secretion-promoting properties of acetylcholine, an appetite-promoting effect can be expected with acetylcholine (46).

Several studies have reported that serum ghrelin and leptin concentrations were associated with cognitive function (47, 48), while another study reported that there is no difference in serum ghrelin and leptin levels between patients with AD and age-matched healthy subjects (49).

As for structural and functional changes in the hypothalamus among patients with MCI and AD, there are limited studies. Ahmed et al. reported that the hypothalamic volumes and appetite-related hormone levels were not different between those with AD and the healthy elderly (49). However, another study reported decreased glucose metabolism in the hypothalamus of those with AD (50). Appetite and nutritional status are also associated with brain lesions other than those in the hypothalamus. Ismail et al. showed an association between hypoperfusion in the medial prefrontal area and appetite loss (51). The medial prefrontal area has been shown to be associated with apathy (52) and nutritional status, including waist to height ratio (53), and weight change (54). Based on these previous studies, it is suggested that nutritional problems including appetite loss among patients with cognitive impairment may be involved in hormonal changes and/or structural and functional brain changes.

### Herbal Medicine for the Treatment of Appetite Loss in Dementia

There are several causes of appetite loss. Although there is no specific medicine against appetite loss, several medications, such as antipsychotic drugs, are expected to have an appetite-improving effect. It has been reported that ghrelin (55) and olanzapine (antipsychotic drug) (56) successfully increased appetite. While most anti-dementia drugs may cause a loss of appetite as a side effect, it is reported that rivastigmine may have an appetite-enhancing effect (57).

Some herbal medicines are also shown to improve appetite and mood. Of these, rikkunshito, hochuekkito, and ninjin'yoeito are often selected for elderly people, especially those with suspected frailty. Citrus unshiu peel and glycyrrhiza, which are crude drug components of herbal medicine, are included in both these drugs. Hesperidin, nobiletin (components of citrus unshiu peel), and isoliquiritigenin (composition of glycyrrhiza) are reported to increase and restore secretion of ghrelin (58). In addition to improving appetite, these herbal medicines may be expected to impact cognitive function. It was suggested that ginseng, hesperidin, and narirutin inhibit amyloid β aggregation and improve learning and memory function (59, 60). In addition, tenuigenin, the main component of polygalae radic, has been shown to promote the proliferation and differentiation of hippocampal neural stem cells. BT-11, an extract from polygalae radic, is reported to improve cognitive function in older adults, and is also licensed as a dietary supplement (61).

Polypharmacy increases with aging and patients with dementia are more likely to have comorbidities and polypharmacy (62). It is reported that polypharmacy is among the causes of sarcopenia as well as of malnutrition and decreased QOL (16). Appetite and gustatory function may be affected by some types of drug (63). Therefore, the use of herbal medicines, which contain various crude ingredients and affect multiple symptoms, may be suggested for the treatment of elderly people. It is important to establish such comprehensive treatment policy for prevention of appetite loss and cognitive impairment as is deemed suitable for older individuals with MCI and early-stage AD.

## Limitations

This study has several limitations. First, because of the cross-sectional design of this study, the cause-effect relationship between sarcopenia and appetite remains unclear. Secondly, appetite and exercise habits were assessed by questionnaire, and actual dietary intake and physical activity were not evaluated. In patients with dementia, self-administered questionnaires are considered less reliable, given their memory dysfunction. However, it has been shown that CNAQ had the same level of validity and reproducibility in those with dementia as in local elderly residents (18). Despite this, however, nutritional status and physical activity are required to be evaluated based on objective measures in future research. Thirdly, we only evaluated the number of drugs patients prescribed to determine the presence of polypharmacy. Thus, we were unable to clarify behind the mechanisms, e.g., effect of individual drugs and/or interaction between drugs, of significant link between polypharmacy and sarcopenia. Finally, some other confounding factors previously reported in sarcopenia, such as inflammation, oxidant stress, and vitamin D were not fully investigated.

## Conclusions

In conclusion, our study demonstrated that sarcopenia was independently associated with low BMI, polypharmacy, and appetite loss in patients with MCI and early-stage AD. Appetite loss is a symptom that can be recognized by people around patients and be improved through pharmacological and non-pharmacological intervention. We believe that the study results offer important clues as to how to suppress the increase of sarcopenia as well as frailty.

## Author Contributions

AK, TaiS, and TakS conceived the study. AK and TaiS contributed to the combined data analysis and presentation of data. SN contributed to the quality control of the clinical data. All authors contributed to the writing of the manuscript and revisions.

### Conflict of Interest Statement

The authors declare that the research was conducted in the absence of any commercial or financial relationships that could be construed as a potential conflict of interest.
